# Endobronchial obstruction in connective tissue diseases: an uncommon but life threatening complication: two case reports

**DOI:** 10.1186/s13256-023-04058-x

**Published:** 2023-08-02

**Authors:** Rushab Shah, Lisa Lim, Mandana Nikpour

**Affiliations:** 1 Department of Rheumatology, St Vincent’s Hospital (Melbourne), 41 Victoria Parade, Fitzroy, VIC 3065 Australia; 2 The University of Melbourne at St Vincent’s Hospital (Melbourne), 41 Victoria Parade, Fitzroy, VIC 3065 Australia

**Keywords:** Airway obstruction, Relapsing polychondritis, Granulomatosis with polyangiitis

## Abstract

**Background:**

Granulomatosis with polyangiitis and relapsing polychondritis are rare, multisystemic and potentially life-threatening connective tissue diseases. We present two cases of severe endobronchial obstruction in the aforementioned conditions and discuss difficulties with detection and treatment. Despite differing underlying pathophysiologies, endobronchial disease is a less frequently reported but serious complication of both conditions.

**Case presentation:**

Case 1, a 31-year-old South Asian woman with relapsing polychondritis, required partial tracheal resection and reconstruction in combination with immunosuppressive therapy to achieve respiratory recovery following collapse of her right main bronchus and a stricture in her left main bronchus. Case 2, a 22-year-old Caucasian male with granulomatosis with polyangiitis, underwent surgical resection of an endobronchial growth causing occlusion of his right main bronchus. Although his respiratory status was initially stabilised with increased immunosuppression, he continues to have disease progression in spite of this.

**Conclusions:**

Our cases highlight the importance of a multidisciplinary approach combining immunosuppression with supportive care and judicious use of surgical interventions in select cases. A further review of the literature shows endobronchial obstruction is potentially under-reported due to overlap in connective tissue disease symptomatology and there is no consensus on best practice.

## Introduction

Granulomatosis with polyangiitis (GPA) and relapsing polychondritis (RP) are rare, multisystemic and potentially life-threatening connective tissue diseases (CTDs) [1, 2]. RP presents as recurrent episodes of cartilaginous inflammation whereas GPA is a necrotising vasculitis often with formation of granulomas [1, 3]. Despite differing underlying pathophysiologies, endobronchial disease is a less frequently reported but potentially serious complication of both conditions [4].

## Case 1

A 31-year-old South Asian female presented to hospital with a 6-week history of bilateral otalgia, nasal bridge tenderness and painful injected eyes. On initial presentation for ophthalmologic examination, she was found to have bilateral acute anterior non-necrotising scleritis on slit-lamp examination. She was prescribed corticosteroid eye drops and discharged home, where she developed systemic symptoms of fevers, weight loss of 2 kg in 1 month and night sweats. Additionally, she noted dysphonia and occasional dry cough. On later examination, she was noted to have a saddle nose deformity and tender inflamed ears. Initial investigations revealed negative auto-antibodies and elevated inflammatory markers with a C-reactive protein of 150 mg/l and erythrocyte sedimentation rate of 120 mm/h. A plain chest X-ray showed prominence of bilateral hila and widening of paratracheal stripe.

**Fig. 1:**
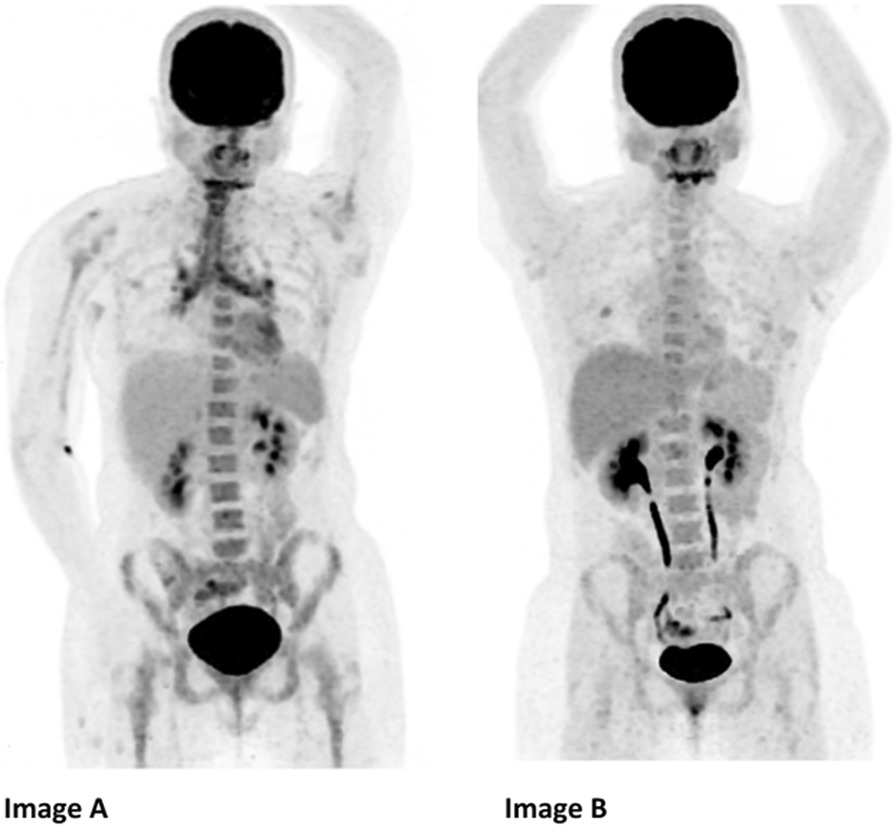
Positron emission tomography scan of Case 1 at diagnosis and following treatment. **A** Positron emission tomography scan at diagnosis showing significant tracheal and endobronchial avidity. **B** Positron emission tomography scan following treatment with adalimumab demonstrating resolution in tracheal and endobronchial avidity

A diagnostic positron emission tomography (PET) scan demonstrated fluorodeoxyglucose (FDG) avidity in her hypopharynx, trachea and bilateral hila (Fig. 1). A clinical diagnosis of relapsing polychondritis was made and she was treated with 1 g intravenous methylprednisolone daily for 3 days, followed by 20 mg oral prednisolone and oral methotrexate (20 mg weekly).

In the months following her diagnosis, she developed progressive shortness of breath and stridor. Computerised tomography (CT) imaging showed tracheomalacia and left main bronchus collapse. At this time, intravenous cyclophosphamide therapy (1 g monthly for 6 months) was added to her treatment regimen.

Eighteen months after her initial presentation, she was admitted for surgical management of her endobronchial disease, as the clinical response to cyclophosphamide was deemed inadequate, with ongoing stridor.

An initial bronchoscopy showed collapse of the right main bronchus and a fixed stricture of the left main bronchus. A subsequent bronchoscopy showed a severe subglottic stricture, requiring dilatation. Following this, the patient’s respiratory status deteriorated significantly. It was thought that active RP was contributing to airway compromise, with bronchoscopy causing localised irritation and a flare up of her disease.

A decision was made to proceed with partial tracheal resection and reconstruction. Following this procedure, she continued to fluctuate in her respiratory status, requiring ventilatory support in the intensive care unit (ICU). Through ongoing discussion between the surgical and rheumatology units, her therapy was escalated to intravenous corticosteroids with the addition of tumour necrosis factor alpha inhibition (TNFi) with adalimumab 40 mg subcutaneously fortnightly. The initial hesitation surrounding increasing immunosuppressive therapy was due to wound healing concerns. Following immunosuppressive therapy escalation, the patient finally achieved respiratory recovery and her disease remains controlled on TNFi therapy.

## Case 2

A 22-year-old Caucasian male with GPA was admitted to ICU with respiratory failure requiring invasive ventilation. In the hours prior, he reported sudden onset dyspnoea and haemoptysis. This was in the context of anticoagulation with apixaban for bilateral provoked pulmonary emboli, which were found incidentally 6-months prior.

GPA had been diagnosed seven months before this presentation, with a subacute presentation of rhinitis and otitis media. In the time since, his manifestations evolved to include: bilateral facial nerve palsies, chronic sinusitis, a cavitating lung lesion and likely renal involvement with significant haematuria and proteinuria but no renal biopsy given his anti-coagulation. On diagnosis, he was found to be cANCA positive with proteinase 3 specificity (233 IU/ml, normal < 2 IU/ml). A sinus mucosal biopsy was performed with histology showing giant cells and necrosis. Initial induction therapy consisted of 1 g intravenous methylprednisolone for 3 days and 750 mg rituximab weekly for four weeks. However, as subsequent therapy with azathioprine was unable to stabilise his disease, cyclophosphamide was commenced.

At the time of admission to ICU, he had received only a single dose of cyclophosphamide (1.2 g intravenous). CT of the chest showed complete obstruction of the right main bronchus and collapse of the right lung. Flexible bronchoscopy was performed revealing an endobronchial growth at the origin of the right main bronchus, causing occlusion. Upon first attempt, the endoscopy was unable to be passed beyond the lesion; no pulmonary haemorrhage was visible. Two days later, a second attempt at bronchoscopy was successful in bronchial dilatation and resection of the mass.

Following resection, the patient was extubated and remained stable on non-invasive ventilation.

A further course of 1 g intravenous methylprednisolone was administered, followed by the second infusion of 1.2 g intravenous cyclophosphamide. Additionally, seven rounds of plasmapheresis were commenced to control his severe and progressive disease. Despite multiple immunosuppressive agents and surgical interventions, the patient’s disease remains active and he has required further admissions to ICU with progressive airway obstruction.

## Discussion

These cases highlight the life-threatening severity with which endobronchial disease can manifest in CTDs. Early identification of endobronchial disease is vital in the prevention of bronchomalacia and airway stenoses [1, 4].

To evaluate for presence and severity of endobronchial disease, several modalities including lung function testing, endoscopic and imaging tools are employed. FDG PET/CT imaging has been shown to be a useful tool to assess for the presence of endobronchial disease as well as to monitor activity and response to therapy [5, 6]. Dynamic expiratory CT is another non-invasive screening modality that has been demonstrated to be useful in detecting fixed stenoses and dynamic airway collapse [4].

It has been noted that endobronchial disease can develop in patients whilst on systemic immunosuppression and irrespective of disease control in other organs [4, 7].

Treatment of endobronchial complications of CTDs is tailored to the individual patient with no consensus on best practice. Therapies include systemic immunotherapy, surgical and endoscopic procedures [5, 7, 8]. A multi-centre French study, recruiting 81 patients with tracheal and subglottic stenosis included 33 with granulomatosis and polyangiitis (41%) and 21 relapsing polychondritis (26%) [9]. A majority of patients (81%) were managed with glucocorticoids in combination with immunosuppressive agents, with rituximab used mainly in GPA-related stenosis (70%) and cyclophosphamide in relapsing polychondritis (62%) [9]. However, as was seen in Case 1, surgical and/or endoscopic management without concomitant escalation in immunosuppressive treatment is unlikely to result in sustained improvement. Similarly, endoscopic dilatation was required for 56 patients (69%) of patients seen in this study [9].

Case 1 supports the use of TNFi in the management of severe complicated RP and is in keeping with several other case reports. As seen in Case 2, almost all patients with endobronchial involvement in GPA have relapses of their condition requiring re-evaluation of treatment [5]. Both cases highlight the need for a multi-disciplinary approach to management of airway obstruction in CTDs including ventilatory support, immunosuppression, and in some cases surgery.

## Conclusion

Endobronchial disease in CTDs such as RP and GPA is a life-threatening complication. Endobronchial involvement may be more common than reported due to overlap in symptomatology with other manifestations of CTDs and lack of uniform evaluation techniques.

To date, there are no standardised diagnostic or therapeutic approaches for endobronchial complications of CTDs. Relapses in the short and long term in patients with endobronchial disease remain an ongoing management dilemma. Further studies are required to elucidate appropriate diagnostic and treatment modalities.

## Data Availability

Not applicable.

## Abbreviations

RP: Relapsing polychondritis
GPA: Granulomatosis with polyangiitis
CTD: Connective tissue disease
PET: Positron emission tomography
FDG: Fluorodeoxyglucose
CT: Computerised tomography
ICU: Intensive care unit
TNFi: Tumour necrosis factor alpha inhibition
